# Scale development: ten main limitations and recommendations to improve future research practices

**DOI:** 10.1186/s41155-016-0057-1

**Published:** 2017-01-25

**Authors:** Fabiane F. R. Morgado, Juliana F. F. Meireles, Clara M. Neves, Ana C. S. Amaral, Maria E. C. Ferreira

**Affiliations:** 1 Institute of Education, Universidade Federal Rural do Rio de Janeiro, BR-465, km 7, Seropédica, Rio de Janeiro 23890-000 Brazil; 2 Faculty of Psychology, Universidade Federal de Juiz de Fora, Rua José Lourenço Kelmer, s/n—Campus Universitário Bairro São Pedro, Juiz de Fora, Minas Gerais 36036-900 Brazil; 3 Faculty of Physical Education of the Instituto Federal de Educação, Ciência e Tecnologia do Sudeste de Minas Gerais, Av. Luz Interior, n 360, Estrela Sul, Juiz de Fora, Minas Gerais 36030-776 Brazil

**Keywords:** Assessment, Measurement, Psychometrics, Reliability, Validity

## Abstract

The scale development process is critical to building knowledge in human and social sciences. The present paper aimed (a) to provide a systematic review of the published literature regarding current practices of the scale development process, (b) to assess the main limitations reported by the authors in these processes, and (c) to provide a set of recommendations for best practices in future scale development research. Papers were selected in September 2015, with the search terms “scale development” and “limitations” from three databases: Scopus, PsycINFO, and Web of Science, with no time restriction. We evaluated 105 studies published between 1976 and 2015. The analysis considered the three basic steps in scale development: item generation, theoretical analysis, and psychometric analysis. The study identified ten main types of limitation in these practices reported in the literature: sample characteristic limitations, methodological limitations, psychometric limitations, qualitative research limitations, missing data, social desirability bias, item limitations, brevity of the scale, difficulty controlling all variables, and lack of manual instructions. Considering these results, various studies analyzed in this review clearly identified methodological weaknesses in the scale development process (e.g., smaller sample sizes in psychometric analysis), but only a few researchers recognized and recorded these limitations. We hope that a systematic knowledge of the difficulties usually reported in scale development will help future researchers to recognize their own limitations and especially to make the most appropriate choices among different conceptions and methodological strategies.

## Introduction

In recent years, numerous measurement scales have been developed to assess attitudes, techniques, and interventions in a variety of scientific applications (Meneses et al. 2014). Measurement is a fundamental activity of science, since it enables researchers to acquire knowledge about people, objects, events, and processes. Measurement scales are useful tools to attribute scores in some numerical dimension to phenomena that cannot be measured directly. They consist of sets of items revealing levels of theoretical variables otherwise unobservable by direct means (DeVellis 2003).

A variety of authors (Clark and Watson 1995; DeVellis 2003; Nunnally 1967; Pasquali 2010) have agreed that the scale development process involves complex and systematic procedures that require theoretical and methodological rigor. According to these authors, the scale development process can be carried out in three basic steps.

In the first step, commonly referred as “item generation,” the researcher provides theoretical support for the initial item pool (Hutz et al. 2015). Methods for the initial item generation can be classified as deductive, inductive, or a combination of the two. *Deductive* methods involve item generation based on an extensive literature review and pre-existing scales (Hinkin 1995). On the other hand, *inductive* methods base item development on qualitative information regarding a construct obtained from opinions gathered from the target population—e.g., focus groups, interviews, expert panels, and qualitative exploratory research methodologies (Kapuscinski and Masters 2010). The researcher is also concerned with a variety of parameters that regulate the setting of each item and of the scale as a whole. For example, suitable scale instructions, an appropriate number of items, adequate display format, appropriate item redaction (all items should be simple, clear, specific, ensure the variability of response, remain unbiased, etc.), among other parameters (DeVellis 2003; Pasquali 2010).

In the second step, usually referred to as the “theoretical analysis,” the researcher assesses the content validity of the new scale, ensuring that the initial item pool reflects the desired construct (Arias et al. 2014). A content validity assessment is required, since inferences are made based on the final scale items. The item content must be deemed valid to instill confidence in all consequent inferences. In order to ensure the content validity, the researcher seeks other opinions about the operationalized items. The opinions can be those of expert judges (experts in the development scales or experts in the target construct) or target population judges (potential users of the scale), enabling the researcher to ensure that the hypothesis elaborated in the research appropriately represents the construct of interest (Nunnally 1967).

In the last step, psychometric analysis, the researcher should assess whether the new scale has construct validity and reliability. Construct validity is most directly related to the question of what the instrument is in fact measuring—what construct, trait, or concept underlies an individual’s performance or score on a measure (Churchill 1979). This refers to the degree to which inferences can be legitimately made from the observed scores to the theoretical constructs about which these observations are supposed to contain information (Podsakoff et al. 2013). Construct validity can be assessed with the use of exploratory factor analysis (EFA), confirmatory factor analysis (CFA), or with convergent, discriminant, predictive/nomological, criterion, internal, and external validity. In turn, reliability is a measure of score consistency, usually measured by use of internal consistency, test-retest reliability, split-half, item-total correlation/inter-item reliability, and inter-observer reliability (DeVellis 2003). To ensure construct validity and reliability, the data should be collected in a large and appropriately representative sample of the target population. It is a common rule of thumb that there should be at least 10 participants for each item of the scale, making an ideal of 15:1 or 20:1 (Clark and Watson 1995; DeVellis 2003; Hair Junior et al. 2009).

Although the literature on theoretical and methodological care in scale development is extensive, many limitations have been identified in the process. These include failure to adequately define the construct domain, failure to correctly specify the measurement model, underutilization of some techniques that are helpful in establishing construct validity (MacKenzie et al. 2011), relatively weak psychometric properties, applicability to only a single form of treatment or manual, extensive time required to fill out the questionnaire (Hilsenroth et al. 2005), inappropriate item redaction, too few items and participants in the construction and analysis, an imbalance between items that assess positive beliefs and those that assess negative beliefs (Prados 2007), social desirability bias (King and Bruner 2000), among others.

These limitations in the scale development process weaken the obtained psychometric results, limiting the future applicability of the new scale and hindering its generalizability. In this sense, knowledge of the most often reported limitations is fundamental in providing essential information to help develop best practices for future research in this area. The purpose of this article is threefold: (a) to provide a systematic review of the published literature regarding some current practices of the scale development process, (b) to assess the main limitations reported by the authors in this process, and (c) to provide a set of recommendations for best practices in future scale development research.

## Review

### Method

This systematic review identified and selected papers from three databases: Scopus, PsycINFO, and Web of Science. There was no time restriction in the literature search, which was completed in September 1, 2015. The following search term was used: “scale development.” In the set of databases analyzed, the search was done inclusively in “Any Field” (PsycINFO), in “Article Title, Abstract, Keywords” (Scopus), or in any “Topic” (Web of Science). In addition, we used an advanced search to filter the articles in (search within results), with the search term “limitations” identified in “Any Field” in all databases. Both terms were used in English only. Four reviewers evaluated the papers in an independent and blinded way. Any disagreements on eligibility of a particular study were resolved through consensus among reviewers.

**Fig. 1:**
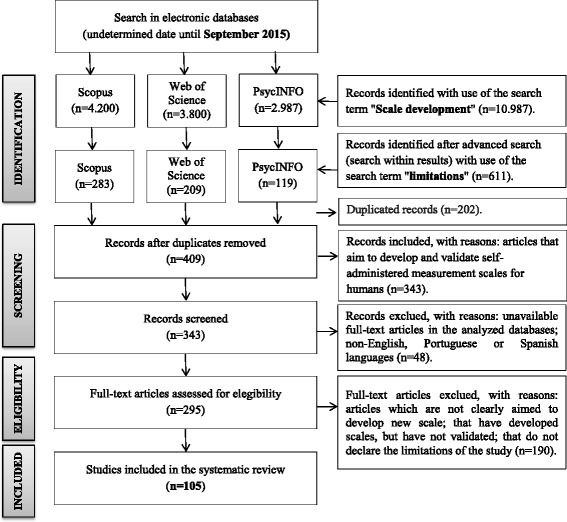
Flowchart showing summary of the systematic process of identifying and selecting article

Figure 1 shows a flowchart summarizing the strategy adopted for identification and selection of studies. We used only one inclusion criteria for the evaluation of the studies: (a) articles that aim to develop and validate self-administered measurement scales for humans. We excluded (a) unavailable full-text papers in the analyzed databases, (b) papers in languages other than English, Portuguese, or Spanish, (c) articles which were not clearly aimed at the development of a new scale (i.e., we excluded articles investigating only the reliability, validity, or revisions of existing scales and studies that describe the validation of instruments for other languages), (d) papers with unvalidated scales, and (e) articles that did not declare the limitations of the study.

### Results

In all, this systematic review evaluated 105 studies published between 1976 and 2015. Most (88.5%) was published between 2005 and 2015, and only two studies date from the last century. We analyzed two major issues: (a) *current practices of the scale development process*—considering the three steps usually reported in the literature (step 1—item generation, step 2—theoretical analysis, step 3—psychometric analysis), the number of participants in step 3, the number of items in the beginning scale, and the number of items in the final scale; (b) m*ain limitations reported by the authors in the scale development process*—considering the limitations observed and recorded by the authors during the scale development process. The description of these results can be found in Table 1.

**Table 1:** Systematic review of the scale development process recorded in 105 included studies

Study	Scale	Step 1	Step 2	Step 3	N step 3	Initial item pool	Final item pool	Main limitations reported
Aagja and Garg (2010)	PubHosQual Scale	LR/ES/I	EJ	EFA/CFA/NV/CV/DV/ICR	401	59	24	LG
Ahmad et al. (2009)	Service Quality Scale	LR/FC	EJ	CFA/CV/DV/S-RR/ICR	413	31	10	LG
Akter et al. (2013)	MHealth Service Quality Scale	LR/ES/FC/I	EJ	EFA/CFA/NV/PV/CV/DV/I-JR/I-T-CR/ICR	305	29	22	LG/CSM
Alvarado-Herrera et al. (2015)	CSRConsPerScale	LR/ES	EJ	CFA/CV/DV/NV/ICR	1087	73	18	LG/Lack of the PV
Armfield (2010)	IDAF-4C^+^	LR/ES	EJ	EFA/CtV/PV/T-RR/ICR	1083	29	8	LG/Lack of the CV/SRM
Atkins and Kim (2012)	Smart Shopping Scale	LR/FC/I	EJ/TPJ	EFA/CFA/NV/CV/DV/ICR	1.474	62	15	LG
Bagdare and Jain (2013)	Retail Customer Experience Scale	LR/EP	EJ/TPJ	EFA/CFA/CV/ICR	676	45	12	LG/This study has not established DV and NV
Bakar and Mustaffa (2013)	Organizational Communication Scale	LR/FC	EJ	EFA/CFA/CtV/CV/DV/T-RR/ICR	596	386	38	LG/Inadequate choose variables to be correlated
Beaudreuil et al. (2011)	URAM	EP/I	EJ/TPJ	EFA/CV/DiV/T-RR/ICR	138	52	9	LG/SSS
Bhattacherjee (2002)	Individual trust in online firms scale	LR/ES	TPJ	CFA/CV/DV/NV/ICR	269	18	7	WBS
Blankson et al. (2007)	International consumers’selection of banks scale	LR/FC	EJ/TPJ	EFA/CFA/CV/PV/NV/ICR/I-T-CR	773	60	18	LG
Blankson et al. (2012)	Scale measuring college students’ choice criteria of credit cards	FC/ES	EJ	EFA/CFA/CV/DV/S-RR/ICR	405	59	19	LG/CSM
Bolton and Lane (2012)	IEO	LR/ES	TPJ	EFA/IV/EV/CV/DV/I-T-CR/ICR	1162	NCR	10	LG/Lack of the CFA
Bova et al. (2006)	HCR	I/FC.	EJ	EFA/T-RR/ICR	99	58	15	LG/Scale was administered in a face-to-face interview/SSS.
Boyar et al. (2014)	CESS	LR	EJ	CFA/DV/ICR	446	140	52	CSM
Brock and Zhou (2005)	OIU	LR/I	EJ	DV/PV/NV/ICR	112	NCR	7	LG
Brun et al. (2014)	Online relationship quality scale	LR, and ES	EJ/TPJ	EFA/CFA/CV/DV/PV/ICR	476	33	21	LG
Butt and Run (2010)	SERVQUAL model scale	LR/EP	EJ	EFA/CFA/CV/DV/ICR	340	17	17	LG
Caro and García (2007)	Perceived service quality in urgent transport service scale	LR/I/ES	EJ/TPJ	EFA/CFA/DV/CV/NV/I-T-CR/ICR	375	68	38	LG/Lack of the CV or DV
Chahal and Kumari (2012)	CPV Scale	LR/ES	EJ/TPJ	EFA/CFA/CV/I-T-CR/ICR	515	32	27	LG
Chen et al. (2009)	Process Orientation Scale	LR/I	EJ	EFA/CFA/CV/DV/I-I-CR/ICR	356	NCR	6	LG/SSS/Lack of the NV
Choi et al. (2011)	Measure of dyspnea severity and related functional limitations	LR/I/EP	EJ/TPJ	EFA/CFA/CV/DiV/T-RR/ICR	608	364	33	CSM
Christophersen and Konradt (2012)	Reflective and formative usability scales	LR/EP/ES	EJ	CFA/CtV/PV/EV/ICR	378	80	54	CSM/SRM
Cicero et al. (2010)	ASI	LR/EP	EJ/TPJ	EFA/CFA/CV/DV/ICR	1281	NCR	29	Items are not reverse-scored
Coker et al. (2011)	IPPR	LR/I	EJ/TPJ	EFA/CFA/CtV/NV/DV/ICR	1200	65	11	LG
Coleman et al. (2011)	B2B service brand identity scale	LR	EJ/TPJ	EFA/CFA/DV/I-T-CR/ICR	210	119	15	LG/Deductive approach to scale development
Colwell et al. (2008)	Measure of service convenience	LR/I	EJ/TPJ	EFA/CFA/CV/DV/NV/ICR	201	30	17	LG/CSM
Cossette et al. (2005)	Caring Nurse–Patient Interactions Scale	LR/ES	EJ	EFA/CV/CtV/ICR	332	121	70	CSM
Dennis and Bocarnea (2005)	Servant leadership assessment instrument	LR/EP	EJ	EFA/CtV/ICR	293	71	42	MD
Devlin et al. (2014)	Fairness measurement scale	LR, and ES	EJ/TPJ	EFA/CFA/CV/DV/NV/ICR	3100	9	8	LG
Dunham and Burt (2014)	Organizational Memory Scale	LR/ES	NCR	EFA/CFA/CV/T-RR/ICR	431	72	21	SRM
Edwards et al. (2010)	STL	FC	TPJ	EFA/CV/CtV/ICR	270	NCR	84	LG
Feuerstein et al. (2005)	Response to work in those with upper extremity pain scale	LR/FC/ES	TPJ	EFA/T-RR/ICR	282	136	136	LG/SSS
Fisher et al. (2014)	Entrepreneurial Success Scale	LR/I	EJ	EFA/CFA/ICR	213	9	4	SSS/Subjective Analysis/SRM
Flight et al. (2011)	Characteristics-based innovation adoption scale	LR	EJ/TPJ	EFA/CFA/ICR/EV/CV/DV/NV/ICR	430	122	43	LG
Forbush et al. (2013)	EPSI	LR	NCR	EFA/CFA/CV/CtV/DV/T-RR/ICR	1528	160	45	LG
Foster et al. (2015)	GNS	LR/ES	NCR	EFA/CFA/ICR	2259	35	33	Lack of the validity
Franche et al. (2007)	RRTW	LR/EP	EJ	EFA/CFA/CrV/PV/IV/EV/ICR	632	NCR	22	SSS/CSM
Gesten (1976)	HRI	LR/EP/ES	EJ	EFA/T-RR/ICR	592	79	54	LG
Gibbons et al. (2013)	MULTIPleS	LR/ES/QER	TPJ	EFA/T-RR/ICR	490	53	22	LG
Gligor and Holcomb (2014)	SCA	LR/ES/I	EJ/TPJ	EFA/CFA/CV/DV/EV/ICR	151	NCR	21	CSM
Glynn et al. (2015)	PFS	ES/QER	NCR	EFA/CV/T-RR/ICR	1496	26	10	LG/MD
Gottlieb et al. (2014)	Consumer perceptions of trade show effectiveness scale	LR/I	NCR	EFA/CFA/CV/DV/NV/I-T-CR/ICR	739	13	11	LG/Items ambiguous/Difficult to control variables
Hall et al. (2002)	General Trust in physicians scale	LR/FC/EP	EJ/TPJ	EFA/CV/CtV/ICR	502	25	11	LG/CSM
Han et al. (2011)	Scale of switching barriers in full-service restaurants	LR/FC	EJ/TPJ	EFA/CFA/CV/NV/I-JR/ICR	401	NCR	17	LG
Henderson-King and Henderson-King (2005)	ACSS	LR	TPJ	EFA/DV/CV/T-RR/ICR	1288	26	15	LG
Hernandez and Santos (2010)	Development-based trust	LR/I	TPJ	EFA/CFA/CV/DV/NV/ICR	238	30	27	CSM
Hildebrandt et al. (2004)	MDDI	LR/ES	NCR	EFA/CV/DiV/T-RR/ICR	245	21	20	LG/Lack of the DV
Ho and Lin (2010)	Scale for measuring internet banking service quality	LR/I/ES	TPJ	EFA/DV/CV/ICR	130	30	17	SSS
Jong et al. (2014)	CRIQ	LR/ES	EJ	EFA/CFA/T-RR/ICR	310	120	120	Lack of the CFA - the CFI fit is below the 0.90
Kim et al. (2011)	CEI	LR	TPJ	EFA/CFA/CV/DV/ICR	397	134	26	LG/Lack of the validity/WBS
Kim et al. (2014)	SAPS	LR	EJ	CFA/CtV/CV/ICR	795	29	15	Lack of the DV
Kwon and Lennon (2011)	Brand Association Scale	LR	EJ	EFA/CFA/CV/DV/I-JR/ICR	671	28	14	LG
Lin and Hsieh (2011)	SSTQUAL Scale	LR/FC/I	EJ	EFA/CFA/CV/DV/NV/I-T-CR/ICR	1238	75	20	LG/subjectivity in the EFA and CFA
Lombaerts et al. (2009)	SRLTB	LR	EJ	EFA/CFA/ICR	952	39	10	Initial unsatisfactory factor analysis output
Lucas-Carrasco et al. (2011)	QOCS	LR/FC	TPJ	EFA/CFA/CV/DV/ICR	3772	44	17	Recruitment of a larger number of interviewers
Mahudin et al. (2012)	Measuring rail passenger crowding	LR/ES	EJ/TPJ	EFA/CFA/CV/DV/ICR	525	9	20	Lack of the CtV/SRM
Medina-Pradas et al. (2011)	BDSEE	ES/EP	EJ	EFA/CV/CtV/ICR	77	14	14	SSS/CSM
Morean et al. (2012)	AEAS	LR/ES	EJ/TPJ	EFA/CFA/CV/CtV/DV/T-RR/ICR	546	40	22	LG/SRM/CSM
Morgado et al. (2014)	SAS-EB	LR/FC	EJ/TPJ	CFA/CV/DV/ICR	318	33	18	Lack of the use of a validated scale in the CV
Nagy et al. (2014)	Scale to measure liabilities and assets of newness after start-up	LR/I	EJ	EFA/CFA/DV/ICR	260	235	19	LG/SSS
Napoli et al. (2014)	Consumer-based brand authenticity scale	LR	EJ/TPJ	EFA/CFA/CV/DV/PV/ICR	762	157	14	Lack of a more robust LR
Negra and Mzoughi (2012)	OCPS	LR/I	EJ	EFA/CFA/CV/DV/NV/I-T-CR/ICR	512	77	5	Widely heterogeneous sample/Brevity of the scale.
Ngorsuraches et al. (2007)	TRUST-Ph	LR/FC/EP/ES	EJ	EFA/CV/CtV/ICR	400	40	30	LG/SSS/MD/social desirability bias
Oh (2005)	Affective reactions to print apparel advertisements scale	LR/FC/ES	TPJ	EFA/CFA/CV/DV/CtV/ICR	128	66	54	LG
Olaya et al. (2012)	ISAD	EP/ES	EJ	CV/DiV/T-RR/ICR	76	20	17	LG
Omar and Musa (2011)	LPSQual	LR/FC	EJ	EFA/CFA/CV/DV/NV/ICR	655	57	26	LG/Lack of the NV/CSM
Pan et al. (2013)	PMGS	I	EJ/TPJ	EFA/CFA/CV/S-RR/I-I-CR/I-T-CR/ICR	554	71	14	LG/SRM/Lack of the T-RR
Patwardhan and Balasubramanian (2011)	Measurement scale for brand romance	LR/ES/QER	TPJ	EFA/CFA/CV/DV/CtV/NV/ICR	711	70	12	LG
Pimentel et al. (2007)	EPM	NCR	EJ/TPJ	EFA/CFA/ICR	480	13	13	LG/Lack of the CV and DV
Pommer et al. (2013)	PCQ	EP/FC	TPJ	EFA/CFA/CV/ICR	953	391	18	CSM
Reed et al. (2011)	ESLS	ES	EJ	EFA/CFA/CV/DV/ICR	218	55	25	LG/SRM/WBS
Rice et al. (2013)	MDRS-22	LR	EJ	EFA/CFA/ICR	1176	82	22	LG/Lack of the T-RR/Lack of the CV
Riedel et al. (2011)	RSM-scale	LR/ES	EJ/TPJ	DV/T-RR/ICR	136	43	36	LG
Rodrigues and Bastos (2012)	Organizational Entrenchment Scale	EP/ES	EJ	EFA/CFA/I-T-CR/I-I-CR/ICR	721	31	22	LG
Rodríguez et al. (2013)	VEDAS	ES	NCR	EFA/CFA/CV/CtV/T-RR/ICR	1034	40	20	Long time between the test and retest/Lower Cronbach’s alpha
Rosenthal (2011)	IEK	EP	EJ	EFA/CV/CrV/I-T-CR/I-I-/T-RR/ICR	292	54	21	LG/SSS
Saxena et al. (2015)	UCLA Hoarding Severity Scale	LR/EP	EJ	EFA/CV/DV/I-I-CR/ICR	127	NCR	10	Lack of the T-RR/Lack of the instructions for raters in the initial version of the scale
Schlosser and McNaughton (2009) -	I-MARKOR scale	LR/FC/I	EJ/TPJ	EFA/CFA/CV/DV/NV/ICR	138	71	20	SSS/CSM.
Sewitch et al. (2003)	PPDS	LR	EJ	EFA/CrV/CV/ICR	200	10	10	LG/CrV was limited/content validity was not formally assessed
Sharma (2010)	Personal Cultural Orientations Scale	LR/I	EJ	EFA/CFA/NV/CV/PV/DV/ICR	2332	96	40	LG/Lack of the PV
Sharma and Gassenheimer (2009)	SPC Scale	LR/EP/I	EJ	EFA/CFA/CV/DV/ICR	511	8	17	Lack of the EV
Shawyer et al. (2007)	VAAS	LR	EJ	CV/T-RR/ICR	41	61	31	Lack of a more robust demonstration of the validity/SSS
Sin et al. (2005)	CRM	LR	EJ	EFA/CFA/CV/DV/NV/ICR	641	78	18	LG/CSM
Sohn and Choi (2014)	Expected Interactivity Scale	LR/EP/I	EJ	EFA/CFA/CV/DV/CtV/T-RR/ICR	378	50	12	Lack of the empirical test
Song et al. (2011)	SECI	LR	EJ	EFA/CFA/CV/I-I-CR/ICR	469	26	17	LG/deductive approach
Staines (2013)	Investigative Thinking Styles Scale	LR	TPJ	EFA/CV/CtV/ICR	545	68	16	LG
Sultan and Wong (2010)	Performance-based servisse quality model scale	LR/FC/ES	EJ	EFA/CFA/ICR	362	67	67	The study uses three sources to collect data
Swaid and Wigand (2009)	E-Service Quality Scale	LR	TPJ	EFA/CFA/CV/DV/ICR.	557	NCR	28	Online survey
Tanimura et al. (2011)	DDLKOS	LR/I	EJ	EFA/CFA/CtV/ICR	362	48	14	Inadequate choose variables to be correlated with that of the study
Taute and Sierra (2014)	Brand Tribalism Scale	LR/ES	NCR	EFA/CFA/CV/DV/ICR	616	35	16	LG
Tombaugh et al. (2011)	SEW	LR/EP	NCR	EFA/CFA/CV/DV/PV/ICR	348	5	5	CSM/Brevity of the scale
Turker (2009)	CSR	LR/FC/ES	TPJ	EFA/I-I-CR/I-T-CR/ICR	269	55	18	LG
Uzunboylu and Ozdamli (2011)	MLPS	LR/I/EP	EJ	EFA/S-RR/ICR	467	31	26	LG
Van der Gaag et al. (2013)	DACOBS	EP	EJ	EFA/CV/ICR/S-RR/T-RR	257	70	42	SSS/Validation performed with patients/Inappropriate choice of the instruments for validation
Von Steinbüchel et al. (2010)	QOLIBRI	LR/ES	EJ	EFA/CFA/T-RR/ICR	2449	148	37	SSS
Voon et al. (2014)	HospiSE	LR/FC	EJ/TPJ	EFA/CFA/CV/DV/CtV/ICR	1558	NCR	21	LG/CSM
Walshe et al. (2009)	DIP	LR/I/ES	TPJ	Ecological validity/ICR	31	48	48	SSS/Lack of the DV, CV and T-RR
Wang and Mowen (1997)	SC	LR	EJ	EFA/CFA/CV/DV/PV/I-T-CR/ICR	140	60	9	SSS
Wepener and Boshoff (2015)	The customer-based corporate reputation of large service organizations scale	LR/ES/FC	EJ	EFA/CFA/NV/CV/DV/ICR	2551	78	19	LG
Williams et al. (2009)	SCSC	LR/I	EJ/TPJ	EFA/CFA/CV/DV/PV/I-T-CR/ICR	162	5	5	LG; b) WBS.
Wilson and Holmvall (2013)	Incivility from customers scale	LR/FC	EJ	EFA/CFA/CV/DV/CtV/ICR	439	27	10	LG/CSM/SRM
Yang et al. (2014)	BLOG-S-INNO Scale	EP	TPJ	EFA/CFA/CV/DV/ICR	498	517	18	LG
Zhang and Hu (2011)	Farmer-buyer relationships in China Scale	LR/ES	EJ/TPJ	EFA/CFA/CV/I-I-CR/ICR	210	39	22	LG
Zheng et al. (2010)	DPEBBS	LR/FC	EJ	EFA/CFA/CtV/T-RR/I-T-CR/ICR	269	51	24	LG/SSS/EFA and CFA - same sample/Reliability coefficients - unsatisfactory.

*N* sample size, *EFA* exploratory factor analysis, *CFA* confirmatory factor analysis, *NV* nomological validity, *CV* convergent validity, *CrV* concurrent validity, *CtV* criterion validity, *DV* discriminant validity, *DiV* divergent validiy, *PV* predictive validity, *IV* internal validity, *EV* external validity, *ICR* internal consistency reliability, *S-RR* split-half reliability, *I-JR* inter-judge reliability, *I-T-CR* item–total correlation reliability, *I-I-CR* inter-item correlation reliability, *T-RR* test-retest reliability, *LR* literature review, *ES* existing scales, *I* interview, *FC* Focus group, *EP* expert panel, *QER* qualitative exploratory research, *NCR* not clearly reported, *EJ* expert judges, *TPJ* target population judges, *LG* limitations of generalization, *SSS* small sample size, *CSM* cross-sectional methodology, *SEM* self-reporting methodology, *WBS* web-based survey, *MD* Missing data

#### Current practices of the scale development process

##### Step 1—item generation

In the first step, 35.2% (*n* = 37) of the studies reported using exclusively deductive methods to write items, 7.6% (*n* = 8) used only inductive methods, and 56.2% (*n* = 59) combined deductive and inductive strategies. The majority of the studies used a literature review (84.7%, *n* = 89) as the deductive method in item generation. In inductive methods, 26.6% of studies (*n* = 28) chose to conduct an interview.

##### Step 2—theoretical analysis

In order to theoretically refine the items, several studies used opinions of experts (74.2%, *n* = 78), whereas others used target population opinions (43.8%, *n* = 46). In addition, 63.8% (*n* = 67) of the studies used only one of these approaches (expert or population judges).

##### Step 3—psychometric analysis

The most common analyses that have been used to assess construct validity are EFA (88.6%, *n* = 93), CFA (72.3%, *n* = 76), convergent validity (72.3%, *n* = 76), and discriminant validity (56.2%, *n* = 59). Most studies opted to combine EFA and CFA (65.7%, *n* = 69). Only 4.7% (*n* = 5) failed to use factor analysis in their research. In relation to study reliability, internal consistency checks were used by all studies and test-retest reliability was the second most commonly used technique (22.8%, *n* = 24).

##### Sample size in step 3 and number of items

Interestingly, 50.4% (*n* = 53) of the studies used sample sizes smaller than the rule of thumb, which is a minimum of 10 participants for each item in the scale. Regarding number of items, the majority of the studies (49.6%, *n* = 52) lost more than 50% of the initial item pool during the validation process.

Table 2 summarizes and provides more details on our findings regarding the current practices in the scale development.

#### Main limitations reported in the scale development process

As result of this systematic review, we found ten main limitations commonly referenced in the scale development process: (1) sample characteristic limitations—cited by 81% of the studies, (2) methodological limitations—33.2%, (3) psychometric limitations—30.4%, (4) qualitative research limitations—5.6%, (5) missing data—2.8%, (6) social desirability bias—1.9%, (7) item limitations—1.9%, (8) brevity of the scale—1.9%, (9) difficulty controlling all variables—0.9%, and (10) lack of manual instructions—0.9%. Table 3 summarizes these findings.

**Table 2:** Summary of current practices of the scale development process

Methods	Number of scales resorting to	Percentage (%) of scales resorting to
Step 1—item generation		
Deductive methods (exclusively)	37	35.2
Inductive methods (exclusively)	8	7.6
Combined deductive and inductive methods	59	56.2
Literature review	89	84.7
Existing scales	40	38
Interviews	28	26.6
Focus groups	25	23.8
Expert panel	23	21.9
Qualitative exploratory research	3	5
Not clearly reported method	1	1
Step 2—theoretical analysis		
Expert judges	78	74.2
Target population judges	46	43.8
Use of just one approach	67	63.8
Combined two approaches	29	27.7
Not clearly reported approach	9	8.5
Step 3—psychometric analysis		
EFA	93	88.6
CFA	76	72.3
Combined EFA and CFA	69	65.7
Lack of EFA and CFA	5	4.7
Convergent/concurrent validity	76	72.3
Discriminant validity	59	56.2
Predictive/nomological validity	34	32.3
Criterion validity	17	16.2
External validity	5	4.7
Internal validity	3	2.8
Internal consistency	105	100
Test-retest reliability	24	22.8
Item-total correlation/inter-item reliability	19	18.1
Split-half reliability	3	2.9
Inter-judge reliability	3	2.9
Sample size about step 3 and number of items		
Sample size smaller than the rule of thumb 10:1	53	50.4
Number of items final scale reduced by 50%	42	40
Number of items final scale reduced more than 50%	52	49.6
Not clearly reported inicial item number	11	10.4

*EFA* exploratory factor analysis, *CFA* confirmatory factor analysis

### Discussion

This systematic review was primarily directed at identifying the published literature regarding current practices of the scale development. The results show a variety of practices that have been used to generate and assess items, both theoretically and psychometrically. We evaluated these current practices, considering three distinct steps (item generation, theoretical analysis, and psychometric analysis). We also considered the relationship between sample size and number of items, since this is considered an important methodological aspect to be evaluated during the scale development process. The results are discussed together with recommendations for best practices in future scale development research.

#### Current practices of the scale development process—findings and research implications

Regarding step 1, item generation, our results show that, although several studies used exclusively deductive methods (e.g., Henderson-King and Henderson-King 2005; Kim et al. 2011), the majority (e.g., Bakar and Mustaffa 2013; Uzunboylu and Ozdamli 2011) combined deductive and inductive methods, a combination consistent with the recommended strategy for the creation of new measures (DeVellis 2003). These findings, however, differ from previous critical reviews of scale development practices, which found that most of the reported studies used exclusively deductive methods (Hinkin 1995; Kapuscinski and Masters 2010; Ladhari 2010). This is particularly important since the quality of generated items depends on the way that the construct is defined. Failing to adequately define the conceptual domain of a construct causes several problems related to poor construct definition, leading to, for example, (a) confusion about what the construct does and does not refer to, including the similarities and differences between it and other constructs that already exist in the field, (b) indicators that may either be deficient or contaminated, and (c) invalid conclusions about relationships with other constructs (MacKenzie et al. 2011). Considering that item generation may be the most important part of the scale development process, future measures should be developed using the appropriate definition of the conceptual domain based on the combination of both deductive and inductive approaches.

Our results suggest that literature review was the most widely used deductive method (e.g., Bolton and Lane 2012; Henderson-King and Henderson-King 2005). This is consistent with the views of several other researchers who have systematically reviewed scales (Bastos et al. 2010; Ladhari 2010; Sveinbjornsdottir and Thorsteinsson 2008). Nevertheless, this finding differs from another study (Kapuscinski and Masters 2010) that found that the most common deductive strategies were reading works by spiritual leaders, theory written by psychologists, and discussion among authors. Literature review should be considered central for the enumeration of the constructs. It also serves to clarify the nature and variety of the target construct content. In addition, literature reviews help to identify existing measures that can be used as references to create new scales (Clark and Watson 1995; DeVellis 2003). In this sense, future research should consider the literature review as the initial and necessary deductive step foundational to building a new scale.

This review also highlights the fact that interviews and focus groups were the most widely used inductive methods (e.g., Lin and Hsieh 2011; Sharma 2010). Similar results were found in the systematic review by Kapuscinski and Masters (2010), Sveinbjornsdottir and Thorsteinsson (2008), and Ladhari (2010). These findings have particular relevance to future researchers, since they emphasize the importance of using methodological strategies that consider the opinions of the target population. Despite the fact that a panel of experts contributes widely to increasing the researchers’ confidence in the content validity of the new scale, it is important to also consider the most original and genuine information about the construct of interest, which can be best obtained through reports obtained from interviews and focus groups with the target population.

Related to step 2, theoretical analysis, the results of this review indicate that expert judges have been the most widely utilized tool for analyzing content validity (e.g., Uzunboylu and Ozdamli 2011; Zheng et al. 2010). Previous studies have also found expert opinion to be the most common qualitative method for the elimination of unsuitable items (Kapuscinski and Masters 2010; Ladhari 2010). In the literature review conducted by Hardesty and Bearden (2004), the authors highlighted the importance of these experts to carefully analyze the initial item pool. They suggested that any research using new, changed, or previously unexamined scale items, should at a minimum be judged by a panel of experts. However, the authors also point out the apparent lack of consistency in the literature in terms of how researchers use the opinions of expert judges in aiding the decision of whether or not to retain items for a scale. Given this inconsistency, the authors developed guidelines regarding the application of different decision rules to use for item retention. For example, the “sumscore decision rule,” defined as the total score for an item across all judges, is considered by the authors to be the most effective in predicting whether an item should be included in a scale and appears, therefore, to be a reasonable rule for researchers to employ.

Future research in developing scales should be concerned, not only with opinions from experts but also with the opinions of the target population. The results of this review show that only a minority of studies considered the review of the scales’ items by members of the target population (e.g., Uzunboylu and Ozdamli 2011; Zheng et al. 2010). In addition, a smaller minority combined the two approaches in the assessment of item content (e.g., Mahudin et al. 2012; Morgado et al. 2014). The limited use of target population opinions is a problem. A previous study of systematic scale development reviews found that the opinion of these people is the basis for content validity (Bastos et al. 2010). As highlighted by Clark and Watson (1995) and Malhotra (2004), it is essential for the new scale to undergo prior review by members of the target population. Pre-test or pilot study procedures make it possible to determine respondents’ opinions of, and reactions to, each item on the scale, enabling researchers to identify and eliminate potential problems in the scale before it is applied at large.

Another problem noted in this systematic review was that some studies failed to clearly report how they performed the theoretical analysis of the items (e.g., Glynn et al. 2015; Gottlieb et al. 2014). We hypothesized that the authors either did not perform this analysis or found it unimportant to record. Future research should consider this analysis, as well as all subsequent analyses, necessary and relevant for reporting.

Almost all studies (95.3%) reported using at least one type of factor analysis—EFA or CFA—in step 3, psychometric analysis (e.g., Sewitch et al. 2003; Tanimura et al. 2011). Clark and Watson (1995) consider that “unfortunately, many test developers are hesitant to use factor analysis, either because it requires a relatively large number of respondents or because it involves several perplexing decisions” (p. 17). They emphasized the importance of the researcher’s need to understand and apply this analysis, “it is important that test developers either learn about the technique or consult with a psychometrician during the scale development process” (Clark and Watson 1995, p. 17). This question seems to have been almost overcome in recent studies, since the vast majority of the analyzed studies used the factor analysis method.

Among the studies than used factor analysis, the majority chose to use EFA (e.g., Bakar and Mustaffa 2013; Turker 2009). Similar to our findings, Bastos et al. (2010) and Ladhari (2010) found EFA to be the more commonly utilized construct validity method when compared to CFA. EFA has extensive value because it is considered to be effective in identifying the underlying latent variables or factors of a measure by exploring relationships among observed variables. However, it allows for more subjectivity in the decision-making process than many other statistical procedures, which can be considered a problem (Roberson et al. 2014).

For more consistent results on the psychometric indices of the new scale, DeVellis (2003) indicates the combined use of EFA and CFA, as was performed with most studies evaluated in this review. In CFA, the specific hypothesized factor structure proposed in EFA (including the correlations among the factors) is statistically evaluated. If the estimated model fits the data, then a researcher concludes that the factor structure replicates. If not, the modification indices are used to identify where constraints placed on the factor pattern are causing a misfit (Reise et al. 2000). Future studies should consider the combined use of EFA and CFA during the evaluation of construct validity of the new measure, and should also apply a combination of multiple fit indices (e.g., modification indices) in order to provide more consistent psychometric results.

After EFA and CFA, convergent validity was the preferred technique used in the vast majority of the studies included in this review (e.g., Brun et al. 2014; Cicero et al. 2010). This finding is consistent with prior research (Bastos et al. 2010). Convergent validity consists in examining whether a scale’s score is associated with the other variables and measures of the same construct to which it should be related. It is verified either by calculating the average variance extracted for each factor when the shared variance accounted for 0.50 or more of the total variance or by correlating their scales with a measure of overall quality (Ladhari 2010). In the sequence of convergent validity, the following methods were identified as favorites in the assessment of construct validity: discriminant validity (the extent to which the scale’s score does not correlate with unrelated constructs) (e.g., Coker et al. 2011), predictive/nomological validity (the extent to which the scores of one construct are empirically related to the scores of other conceptually related constructs) (e.g., Sharma 2010), criterion validity (the empirical association that the new scale has with a gold standard criterion concerned with the prediction of a certain behavior) (e.g., Tanimura et al. 2011), internal (signifies whether the study results and conclusions are valid for the study population), and external validity (generalizability of study) (e.g., Bolton and Lane 2012; Khorsan and Crawford 2014). Considering the importance of validity to ensure the quality of the collected data and the generalized potential of the new instrument, future studies should allow different ways to assess the validity of the new scale, thus increasing the psychometric rigor of the analysis.

With regard to reliability, all studies reported internal consistency statistics (Cronbach’s alpha) for all subscales and/or the final version of the full scale (e.g., Schlosser and McNaughton 2009; Sewitch et al. 2003). These findings are consistent with those of previous review studies (Bastos et al. 2010; Kapuscinski and Masters 2010). DeVellis (2003) explains that internal consistency is the most widely used measure of reliability. It is concerned with the homogeneity of the items within a scale. Given its importance, future studies should to consider alpha evaluation as a central point of measurement reliability, and yet, as much as possible, involve the assessment of internal consistency with other measures of reliability. In the sequence of internal consistency, the following methods were identified by this review: test-retest reliability (analysis of the temporal stability; items are applied on two separate occasions, and the scores could be correlated) (e.g., Forbush et al. 2013), item-total/inter-item correlation reliability (analysis of the correlation of each item with the total score of the scale or subscales/analysis of the correlation of each item with another item) (e.g., Rodrigues and Bastos 2012), split-half reliability (the scale is split in half and the first half of the items are compared to the second half) (e.g., Uzunboylu and Ozdamli 2011), and inter-judge reliability (analysis of the consistency between two different observers when they assess the same measure in the same individual) (e.g., Akter et al. 2013; DeVellis 2003; Nunnally 1967).

Regarding sample size in step 3 and number of items, a particularly noteworthy finding was that most studies utilized sample sizes smaller than the rule of thumb that the minimum required ratio should be 10:1 (e.g., Turker 2009; Zheng et al. 2010). DeVellis (2003) and Hair Junior et al. (2009) comment that the sample size should be as large as possible to ensure factor stability. The ‘observations to variables’ ratio is ideal at 15:1, or even 20:1. However, most of the studies included in this review failed to adopt this rule. Some studies looked for justification on evidence related to the effectiveness of much smaller observations to variables ratios. For example, Nagy et al. (2014) justified the small sample size used in their investigation based on the findings of Barrett and Kline (1981), concluding that the difference in ratios 1.25:1 and 31:1 was not a significant contributor to results obtained in the factor stability. Additionally, Arrindell and van der Ende (1985) concluded that ratios of 1.3:1 and 19.8:1 did not impact the factor stability. Although the rules of thumb vary enormously, ten participants to each item has widely been considered safe recommended (Sveinbjornsdottir and Thorsteinsson 2008).

Finally, several studies had their number final of items reduced by more than 50%. For example, Flight et al. (2011) developed an initial item pool composed of 122 items and finished the scale with only 43. Pommer et al. (2013) developed 391 initial items and finished with only 18. Our findings clearly indicate that a significant amount of items can get lost during the development of a new scale. These results are consistent with previous literature which states both that the initial number of items must be twice the desired number in the final scale, since, during the process of analysis of the items, many may be excluded for inadequacy (Nunnally 1967), and that the initial set of items should be three or four times more numerous than the number of items desired, as a good way to ensure internal consistency of the scale (DeVellis 2003). Future research should consider these issues and expect significant loss of items during the scale development process.

#### Ten main limitations reported in the scale development process—findings and research implications

In addition to identifying the current practices of the scale development process, this review also aims to assess the main limitations reported by the authors. Ten limitations were found, which will be discussed together with recommendations for best practices in future scale development research (Table 3).

**Table 3:** Scale development process—ten main limitations

	Limitations	*n*	%
1	Sample characteristics limitations	85	81
	Homogeneous and/or convenience sample—limitations of generalization	67	64
	Small sample size	18	17
2	Methodological limitations	35	33.2
	Cross-sectional methodology	20	19
	Self-reporting methodology	9	8.5
	Web-based survey	6	5.7
3	Psychometric limitations	32	30.4
	Lack of a more robust demonstration of the construct validity and/or reliability	21	20
	Inadequate choose of the instruments or variables to be correlated with the variable of the study	6	5.7
	Factor analysis limitations	5	4.7
4	Qualitative research limitations	6	5.6
	Deductive approach to scale development	2	1.9
	Lack of a more robust literature review	1	1
	Subjective analysis	1	0.9
	Content validity was not formally assessed	1	0.9
	Recruitment of a larger number of interviewers	1	0.9
5	Missing data	3	2.8
6	Social desirability bias	2	1.9
7	Items limitations	2	1.9
	Items ambiguous or difficult to answer	1	1
	None of the items are reverse-scored	1	0.9
8	Brevity of the scale	2	1.9
9	Difficult to control all variables	1	0.9
10	Lack of a manualized instructions	1	0.9

##### Sample characteristic limitations

The above-mentioned limitations were recorded in the majority of the studies, in two main ways. The first and the most representative way was related to the sample type. Several studies used homogeneous sampling (e.g., Forbush et al. 2013; Morean et al. 2012), whereas others used convenience sampling (e.g., Coker et al. 2011; Flight et al. 2011). Both homogeneous and convenience samples were related to limitations of generalization. For example, Atkins and Kim (2012) pointed out that “the participants for all stages of the study were US consumers; therefore, this study cannot be generalized to other cultural contexts.” Or indeed, “convenience samples are weaknesses of this study, as they pose generalizability questions,” as highlighted by Blankson et al. (2012). Nunnally (1967) suggested that, to extend the generalizability of the new scale, sample diversification should be considered in terms of data collection, particularly in the psychometric evaluation step. Future studies should consider this suggestion, recruiting heterogeneous and truly random samples for the evaluation of construct validity and the reliability of the new measure.

The second way was related to small sample size. As previously described, most of the analyzed studies utilized sample sizes less than 10:1. Only some of the authors recognized this flaw. For example, Nagy et al. (2014) reported that “the sample size employed in conducting the exploratory factor analysis is another potential limitation of the study,” Rosenthal (2011) described, “the current study was limited by the relatively small nonprobability sample of university students,” and Ho and Lin (2010) recognized that “the respondent sample size was small.” Based in these results, we emphasize that future research should seek a larger sample size (minimum ratio of 10:1) to increase the credibility of the results and thus obtain a more exact outcome in the psychometric analysis.

##### Methodological limitations

Cross-sectional methods were the main methodological limitations reported by other studies (e.g., Schlosser and McNaughton 2009; Tombaugh et al. 2011). Data collected under a cross-sectional study design contains the typical limitation associated with this type of research methodology, namely inability to determine the causal relationship. If cross-sectional methods are used to estimate models whose parameters do in fact vary over time, the resulting estimation may fail to yield statistically valid results, fail to identify the true model parameters, and produce inefficient estimates (Bowen and Wiersema 1999). In this way, different authors (e.g., Akter et al. 2013; Boyar et al. 2014) recognized that employing instruments at one point in time limits the ability to assess causal relationships. With the goal of remediating these issues and gaining a deeper understanding of the construct of interest, different studies (e.g., Morean et al. 2012; Schlosser and McNaughton 2009) suggest conducting a longitudinal study during the scale development. Using the longitudinal studies in this process may also allow the assessment of the scale’s predictive validity, since longitudinal designs evaluate whether the proposed interpretation of test scores can predict outcomes of interest over time. Therefore, future studies should consider the longitudinal approach in the scale development, both to facilitate greater understanding of the analyzed variables and to assess the predictive validity.

Self-reporting methodologies were also cited as limitations in some studies (e.g., Fisher et al. 2014; Pan et al. 2013). Mahudin et al. (2012) clarified that the self-reporting nature of quantitative studies raises the possibility of participant bias, social desirability, demand characteristics, and response sets. Such possibilities may, in turn, affect the validity of the findings. We agree with the authors’ suggestion that future research may also incorporate other objective or independent measures to supplement the subjective evaluation of the variables studied in the development of the new scale and to improve the interpretation of findings.

In addition, web-based surveys were another methodological limitation reported in some studies (e.g., Kim et al. 2011; Reed et al. 2011). Although this particular method has time- and cost-saving elements for data collection, its limitations are also highlighted. Researchers have observed that important concerns include coverage bias (bias due to sampled individuals not having—or choosing not to access—the Internet) and nonresponse bias (bias due to participants of a survey differing from those who did not respond in terms of demographic or attitudinal variables) (Kim et al. 2011). Alternatives to minimize the problem in future research would be in-person surveys or survey interviews. Although more costly and more time consuming, these methods reduce problems related to concerns about confidentiality and the potential for coverage and nonresponse bias (Reed et al. 2011). Therefore, whenever possible, in-person surveys or survey interviews should be given priority in future research rather than web surveys.

##### Psychometric limitations

Consistent with previous reports (MacKenzie et al. 2011; Prados 2007), this systematic review found distinct psychometric limitations reported in the scale development process. The lack of a more robust demonstration of construct validity and/or reliability was the most often mentioned limitation in the majority of the analyzed studies. For example, Alvarado-Herrera et al. (2015) reported the lack of a more robust demonstration of the predictive validity whereas Kim et al. (2011) of the nomological validity. Caro and Garcia (2007) noted that the relationships of the scale with other constructs were not analyzed. Saxena et al. (2015) and Pan et al. (2013) described the lack of demonstrable temporal stability (e.g., test-retest reliability). Imprecise or incomplete psychometric procedures that are employed during scale development are likely to obscure the outcome. Therefore, it is necessary for future research to consider adverse consequences for the reliability and validity of any construct, caused by poor test-theoretical practices. Only through detailed information and explanation of the rationale for statistical choices can the new measures be shown to have sufficient psychometric adjustments (Sveinbjornsdottir and Thorsteinsson 2008).

Additionally, the inadequate choice of the instruments or variables to be correlated with the variable of interest was another psychometric limitation cited in some studies (e.g., Bakar and Mustaffa 2013; Tanimura et al. 2011). This kind of limitation directly affects the convergent validity, which is a problem since, as has already been shown in this review, this type of validity has been one of the most recurrent practices in scale development. One hypothesis for this limitation may be the lack of gold standard measures to assess similar constructs as those of a new scale. In such cases, a relatively recent study by Morgado et al. (2014) offers a valid alternative. The authors used information collected on sociodemographic questionnaires (e.g., level of education and intensity of physical activity) to correlate with the constructs of interest. Future researchers should seek support from the literature on the constructs that would be theoretically associated with the construct of interest, searching for alternatives in information collected on, for example, sociodemographic questionnaires, to assess the convergent validity of the new scale.

Another psychometric limitation reported in some studies was related to factor analysis. These limitations were identified in five main forms: (1) EFA and CFA were conducted using the data from the same sample (Zheng et al. 2010)—when this occurs, good model fit in the CFA is expected, as a consequence, the added strength of the CFA in testing a hypothesized structure for a new data set based on theory or previous findings is lost (Khine 2008); (2) lack of CFA (Bolton and Lane 2012)—if this happens, the researcher loses the possibility of assigning items to factors, testing the hypothesized structure of the data, and statistically comparing alternative models (Khine 2008); (3) a certain amount of subjectivity was necessary in identifying and labeling factors in EFA (Lombaerts et al. 2009)—since a factor is qualitative, it is common practice to label each factor based on an interpretation of the variables loading most heavily on it; the problem is that these labels are subjective in nature, represent the authors’ interpretation, and can vary typically from 0.30 to 0.50 (Gottlieb et al. 2014; Khine 2008); (4) the initial unsatisfactory factor analysis output (Lombaerts et al. 2009); and (5) lack of a more robust CFA level (Jong et al. 2014) taken together—when the study result distances itself from statistical results expected for EFA (e.g., KMO, Bartlett test of sphericity) and/or CFA (e.g., CFI, GFI, RMSEA), it results in an important limitation, since the tested exploratory and theoretical models are not considered valid (Khine 2008). Taking these results, future studies should consider the use of separate samples for EFA and CFA, the combination of EFA and CFA, the definition of objective parameters to label factors, and about the consideration for unsatisfactory results of EFA and CFA, seeking alternatives to better fit the model.

##### Qualitative research limitations

This review also found reported limitations on the qualitative approach of the analyzed studies. The first limitation was related to the exclusive use of the deductive method to generate items. It is noteworthy that, although most of the studies included in this review used exclusively deductive methods to generate items, only two studies recognized this as a limitation (Coleman et al. 2011; Song et al. 2011). Both studies used only the literature review to generate and operationalize the initial item pool. The authors recognized the importance of this deductive method to theoretically operationalize the target construct, but they noted that, “for further research, more diverse views should be considered to reflect more comprehensive perspectives of human knowledge-creating behaviors to strengthen the validity of the developed scales” (Song et al. 2011, p. 256) and, “a qualitative stage could have been used to generate additional items […]. This could also have reduced measurement error by using specific language the population used to communicate” (Coleman et al. 2011; p. 1069). Thus, the combination of deductive and inductive approaches (e.g., focus groups or interviews) in item generation is again suggested in future research.

In addition, it is also necessary that the researcher consider the quality of the reviewed literature. Napoli et al. (2014, p. 1096) reported limitations related to the loss of a more robust literature review, suggesting that the scale developed in the study may have been incorrectly operationalized: “Yet some question remains as to whether cultural symbolism should form part of this scale. Perhaps the way in which the construct was initially conceptualized and operationalized was incorrect.” The incorrect operation of the construct compromises the psychometric results of scale and its applicability in future studies.

Another limitation involves the subjective analysis of the qualitative research. Fisher et al. (2014, p. 488) pointed out that the qualitative methods (literature reviews and interviews) used to develop and conceptualize the construct were the main weaknesses of the study, “this research is limited by […] the nature of qualitative research in which the interpretations of one researcher may not reflect those of another.” The authors explained that, due to the potential for researcher bias when interpreting data, it has been recognized that credible results are difficult to achieve. Nevertheless, subjective analysis is the essence and nature of qualitative studies. Some precautions in future studies can be taken to rule out potential researcher bias, such as attempts at neutrality. This is not always possible, however, and this limitation will remain a common problem in any qualitative study.

In turn, Sewitch et al. (2003, p. 260) reported that failure to formally assess content validity was a limitation. The reason given was budgetary constraints. It is worthwhile to remember that the content validity is an important step to ensure confidence in any inferences made using the final scale form. Therefore, it is necessarily required in any scale development process.

An additional limitation was reported by Lucas-Carrasco et al. (2011) in the recruitment of a larger number of interviewers, which may have affected the quality of the data collected. In order to minimize this limitation, the authors reported, “all interviewers had sufficient former education, received training on the study requirements, and were provided with a detailed guide” (p. 1223). Future studies planning the use of multiple interviewers should consider potential resulting bias.

##### Missing data

In connection, missing data was another issue reported by some studies included in this systematic review (e.g., Glynn et al. 2015; Ngorsuraches et al. 2007). Such limitations typically occur across different fields of scientific research. Missing data includes numbers that have been grouped, aggregated, rounded, censored, or truncated, resulting in partial loss of information (Schafer and Graham 2002). Collins et al. (2001) clarified that when researchers are confronted with missing data, they run an increased risk of reaching incorrect conclusions. This is because missing data may bias parameter estimates, inflate type I and type II error rates, and degrade the performance of confidence intervals. The authors also explained that, “because a loss of data is nearly always accompanied by a loss of information, missing values may dramatically reduce statistical power” (p. 330). Therefore, future researchers who wish to mitigate these risks during the scale development must pay close attention to the missing data aspect of the analysis and choose their strategy carefully.

Statistical methods to solve the problem of missing data have improved significantly, as demonstrated by Schafer and Graham (2002), although misconceptions still remain abundant. Several methods to deal with missing data were reviewed, issues raised, and advice offered for those that remain unresolved. Considering the fact that a more detailed discussion of the statistics dealing with missing data is beyond of the scope of this article, more details about missing data analysis can be found in Schafer and Graham (2002).

##### Social desirability bias

Another limitation reported in some studies (Bova et al. 2006; Ngorsuraches et al. 2007) and identified in this systematic review is social desirability bias. This type of bias is considered to be a systematic error in self-reporting measures resulting from the desire of respondents to avoid embarrassment and project a favorable image to others (Fisher 1993). According to King and Bruner (2000), social desirability bias is an important threat to the validity of research employing multi-item scales. Provision of socially desirable responses in self-reported data may lead to spurious correlations between variables, as well as the suppression or moderation of relationships between the constructs of interest. Thus, one aspect of scale validity, which should be of particular concern to researchers, is the potential threat of contamination due to social-desirability response bias. To remedy this problem, we agree with the authors that it is incumbent upon researchers to identify situations in which data may be systematically biased toward the respondents’ perceptions of what is socially acceptable, to determine the extent to which this represents contamination of the data, and to implement the most appropriate methods of control. Details on methods for identifying, testing for, and/or preventing social desirability bias are beyond the scope of this article, but can be found at King and Bruner (2000).

##### Item limitations

In comparison with at least one previous study (Prados 2007), our findings reflect some potential item limitations. Firstly, items that were ambiguous or difficult to answer were the main weaknesses reported by Gottlieb et al. (2014). On this issue, the literature dealing with the necessary caution in wording the items is extensive. For example, items must clearly define the problem being addressed, must be as simple as possible, express a single idea, and use common words that reflect the vocabulary level of the target population. Items should not be inductors or have alternative or underlying assumptions. They must be free of generalizations and estimates, and be written to ensure the variability of responses. In writing the items, the researcher should avoid using fashionable expressions and colloquialisms or other words or phrases that impair understanding for groups of varying ages, ethnicities, religions, or genders. Furthermore, the items should be organized properly. For example, the opening questions should be simple and interesting to win the trust of the subjects. The most delicate, complex, or dull questions should be asked at the end of the sequence (Clark and Watson 1995; Malhotra 2004; Pasquali 2010).

Furthermore, Cicero et al. (2010) reported that the main limitation of their study was the fact that none of the items were reverse-scored. Although some methodologists claim that reverse scoring is necessary to avoid acquiescence among participants, this advice should be taken with caution. There are reports that the reverse-scored items may be confusing to participants, that the opposite of a construct reverse-scored may be fundamentally different than the construct, that reverse-scored items tend to be the worst fitting items in factor analyses, or that the factor structure of scales includes a factor with straightforward wording compared to a reverse-scored factor (Cicero et al. 2010). Awareness of these issues is necessary for future researchers to choose between avoiding acquiescence among participants or preventing a number of other problems related to the use of reverse scores.

##### Brevity of the scale

Limitations on the scale size were also identified in this review. Studies by Negra and Mzoughi (2012) and Tombaugh et al. (2011) mentioned the short version of the scale as their main limitation. In both studies, the final version of the new scale included only five items. Generally, short scales are good, because they require less time from respondents. However, very short scales can in fact seriously compromise the reliability of the instrument (Raykov 2008). To the extent that the researcher removes items of the scale, the Cronbach’s alpha tends to decrease. It is valuable to remember that the minimum acceptable alpha should be at least 0.7, while an alpha value between 0.8 and 0.9 is considered ideal. Scales with many items tend to be more reliable, with higher alpha values (DeVellis 2003). In this context, future researchers should prioritize scales with enough items to keep the alpha within the acceptable range. Although many items may be lost during theoretical and psychometric analysis, an alternative already mentioned in this study would be to begin the initial item pool with at least twice the desired items of the final scale.

##### Difficulty controlling all variables

In addition to all limitations reported, Gottlieb et al. (2014) mentioned a common limitation in different research fields—the difficulty of controlling all the variables that could influence the central construct of the study. The authors reported that “it may be that there are other variables that influence visitors’ perception of trade show effectiveness that were not uncovered in the research” and suggest “future research might yield insights that are not provided here” (p. 104). The reported limitation calls attention to the importance of the first step—item generation—in the scale development process. A possible remedy to this issue would be to know the target construct in detail during the item generation, allowing for all possible and important variables to be investigated and controlled. However, this is not always possible. Even using inductive and deductive approaches to generate items (literature review and interview), the authors still reported that limitation. In this light, future researchers must use care in hypothesizing and testing potential variables that could be controlled during construction of the scale development process.

##### Lack of manual instructions

Finally, this review found a weakness reported on the loss of manualized instructions that regulate the data analysis. Saxena et al. (2015, p. 492) pointed out that the initial version of the new scale “did not contain manualized instructions for raters, so it lacked objective anchor points for choosing specific ratings on many of its questions”. Therefore, an important detail that should have the attention of future researchers are instructions that determine the application methods of the new scale. Pasquali (2010) suggests that when drafting the instructions, the researcher should define the development of operational strategies that will enable the application of the instrument and the format in which it will be presented and decide both how the subject’s response will be given for each item and the way that the respondent should answer each item. The researcher should also define how the scale scores would be analyzed. In addition, the instructions need to be as short as possible without confusion to the subjects of the target population, should contain one or more examples of how the items should be answered, and should ensure that the subject is free of any related tension or anxiety.

### Study limitations and strengths

This review itself is subject to some limitations that should be taken into consideration. First, during the selection of the articles included in the analysis, we may have missed some studies that could have been identified by using other terms related to “scale development.” This may have impacted our findings. However, application of this term alone was recommended by its widespread use by researchers in the area (Clark and Watson 1995; DeVellis 2003; Hinkin 1995; Nunnally 1967) and by the large number of publications identified with this descriptor in the period evaluated, as compared with those screened with correlates (e.g., “development of questionnaire” and “development of measure”). In the same way, we may also have missed numerous studies that, despite recording their weaknesses, did not have the search term “limitations” indexed in the analyzed databases. We could have reduced this limitation by also using the search term ‘weakness’ or a similar word for selection and inclusion of several other articles. However, a larger number of included studies would hinder the operationalization of our findings.

Second, particularly regarding analysis of items and reliability, we lost information about the basic theories that support the scale development process: classical test theory (CTT)—known as classical psychometry—and item response theory (IRT)—known as modern psychometry (PASQUALI 2010). Although it was beyond the scope of this article to examine these theories, information on the employability of one or the other could contribute to a deeper understanding of their main limitations. Future studies could focus on CTT and IRT, compare the applicability of both, and identify their main limitations in the scale development process.

Still, our review is current with studies published until September 2015. As new evidence emerges on current practices and limitations reported in the scale development process, revisions to this systematic review and practice guideline would be required in future studies.

Despite its weaknesses, the strengths of this study should be highlighted. First, this study reviews the updated and consistent literature on scale development practices to be applied in, not only a specific field of knowledge as carried out in most systematic review studies, but across various fields. With this variety of conceptions, we hope to assist future researchers in different areas of human and social sciences in making the most appropriate choice between strategies.

Second, this study differs from most studies of scale development revision, since it primarily considers the conceptions of the authors themselves about the main difficulties and mistakes made during the scale development process in their own studies. We hope to contribute to the efforts of future researchers, based on the knowledge of previous mistakes. While several weaknesses in scale development research were identified, specific recommendations for future research relevant to particular previously dimensions discussed were embedded within the appropriate sections throughout the article.

We observe that, although some weaknesses have been clearly identified in the scale development practices of many studies, only a few researchers recognized and recorded these limitations. This was evidenced in the large number of studies using exclusively deductive approaches to generate the initial item pool and the limited number of studies that recognized this as a limitation, or there were a large number of studies using smaller sample sizes than recommended in the literature for psychometric analysis and the limited number of studies that reported this issue as a limitation. Considering the observed distance between the limitation and its recognition, it is important that future researchers are comfortable with the detailed process of developing a new measure, especially as it pertains to avoiding theoretical and/or methodological mistakes, or at least, if they occur, to mention them as limitations.

## Conclusions

In conclusion, the present research reviews numerous studies that both proposed current practices of the scale development process and also reported its main limitations. A variety of conceptions and methodological strategies and ten mains limitations were identified and discussed along with suggestions for future research. In this way, we believe that this paper makes important contributions to the literature, especially because it provides a comprehensive set of recommendations to increase the quality of future practices in the scale development process.
