# Erratum to: Scale development: ten main limitations and recommendations to improve future research practices

**DOI:** 10.1186/s41155-017-0059-7

**Published:** 2017-03-03

**Authors:** Fabiane F. R. Morgado, Juliana F. F. Meireles, Clara M. Neves, Ana C. S. Amaral, Maria E. C. Ferreira

**Affiliations:** 1 Institute of Education, Universidade Federal Rural do Rio de Janeiro, BR-465, km 7, Seropédica, Rio de Janeiro 23890-000 Brazil; 2 Faculty of Psychology, Universidade Federal de Juiz de Fora, Rua José Lourenço Kelmer, s/ n—Campus Universitário Bairro São Pedro, Juiz de Fora, Minas Gerais 36036-900 Brazil; 3 Faculty of Physical Education of the Instituto Federal de Educação, Ciência e Tecnologia do Sudeste de Minas Gerais, Av. Luz Interior, n 360, Estrela Sul, Juiz de Fora, Minas Gerais 36030-776 Brazil

## Erratum

The original publication [1] contains an error in Table 1.

The correct version:

Medina-Pradas et al. (2011): “EFA/CV/CtV/ICR”

## References

[1] Scale development: ten main limitations and recommendations to improve future research practices *Psicologia: Reflexão e Crítica* Psychology: Research and Review 2017 30:3 DOI: 10.1186/s41155-016-0057-1 10.1186/s41155-016-0057-1 PMC6966966 32025957

